# Effects of auditory processing training on speech perception and brainstem plastisity in adolescents with autism spectrum disorders

**DOI:** 10.22037/ijcn.v15i2.22037

**Published:** 2021

**Authors:** Maryam RAMEZANI, Yones LOTFI, Abdollah MOOSSAVI, Enayatollah BAKHSHI

**Affiliations:** 1 Department of Audiology, University of Social Welfare and Rehabilitation Sciences, Tehran, Iran; 2 Department of Otolaryngology and Head and Neck Surgery, School of Medicine, Iran University of Medical Sciences, Tehran, Iran; 3 Department of Biostatistics, University of Social Welfare and Rehabilitation Sciences, Tehran, Iran

**Keywords:** Autism, Auditory Processing Training, Speech Perception, Plasticity

## Abstract

**Objective:**

Autism spectrum disorder (ASD) is a neurodevelopmental disorder. A major problem of ASD is speech perception impairment in the presence of background noise. Additionally, researchers have reported temporal auditory processing impairment in patients with ASD. In the present study, we evaluated the effects of a temporal-based training program on improvement of speech perception in the presence of noise using the speech auditory brainstem response (sABR).

**Materials & Methods:**

Twenty-eight adolescents with high functional ASD with the mean age of 14.35±1.86 years were randomly selected and divided into an ASD group (11 males and three females) and a control group (13 males and one female). All the subjects had a normal hearing and intelligence threshold and had no history of neurological disorder.

A speech perception test was performed in signal-to-noise ratios of 0 and +10. The intervention group received a temporal processingbased auditory training program, and the control group received a conventional training program. A P-value of <0.05 was considered significant.

**Results:**

After training, speech perception in the presence of noise was significantly higher (P <0.001) and the latency of all sABR waves was lower in the intervention group compared to the control group.

**Conclusion:**

Improvement of speech perception in noisy environments and the reduced latency of sABR waves following a temporal processingbased training program highlight the role of brainstem neural plasticity in speech processing.

## Introduction

Autism spectrum disorder (ASD) is a neurodevelopmental disorder with a higher frequency in boys than girls (4:1). The prevalence of this disorder in 2014 was reported one in 88 children in the United States (1). One of the main problems of children with ASD is impaired speech perception in the presence of background noise (2), which significantly affects communication skills (3).

In everyday life, we are exposed to a variety of environmental noises and the ability to communicate in the presence of noise is an essential skill for successful participation in educational and social environments (2). A spoken signal includes temporal signals such as rapid changes in duration, silent intervals, and rapid rises and drops in amplitude (4). Recognition of these temporal features of spoken signals leads to comprehension of linguistic symptoms, recognition of vowels from consonants, identification of specific consonants, and deduction of subtle signs (5). Therefore, the ability to decode speech to extract meaningful linguistic information is dependent on normal auditory processing. 

Many researchers have reported normal or abnormal temporal processing in children with ASD. In addition, disturbances in the encryption and understanding of the temporal aspects of auditory stimuli (such as duration of stimuli and intervals between successive stimuli) have been reported in this group of children. Since temporal processing plays a significant role in speech perception in noisy environments, the problem of speech comprehension in noisy conditions in children with ASD can be due to a defect in temporal processing (6). 

Numerous studies have shown neural plasticity in auditory pathways in animals and humans (7, 8). Evidence has shown that training programs could contribute to neurophysiological changes and improved hearing skills. Tallal et al. (1981) and Merzenich et al. (1996) who developed a bottom-up auditory-training program showed in their studies that by increasing the difficulty level of exercises, the individual's temporal auditory skills gradually improved (9, 10). Therefore, if the impaired rapid processing of an auditory stimulus causes linguistic and verbal problems, bottom-up interventions, which emphasize the speed of stimulus processing, help to improve these problems. In the study of Song et al. in 2008, a pitch-based training program improved phase locking in the frequency following response (FFR) (8). The effect of training and education on subcortical neural plasticity has been rarely investigated in ASD patients. 

In the present study, brainstem-evoked potentials with a speech stimulus were used to study and compare changes in the amplitude and duration of brainstem evoked potential before and after training. The aim was to determine the potential electrophysiological impact of auditory training interventions. Changes in this test can be considered an objective reason for changing the function of the nervous system. Since Speech ABR is a desirable test for assessing bottom-up hearing processes involved in speech perception in the presence of background noise, the clear link between a stimulus and brainstem responses allows for direct comparison between the frequency and temporal components of the stimulus and the responses (2, 11, 12). The brainstem auditory response to the syllable /da/, which is known as a speech brainstem response or sABR, includes a response with two general categories, the source class and the filter class. The source class includes D, E, and F waves, which show vocal cord vibration. The filter class includes V, A, C, and O waves. The V and A waves indicate the start of sound processing in the brainstem, the C wave indicates a response to the start of a vowel, and the O wave indicates the end of a sound (13, 14).

Regarding the role of temporal auditory processing in speech perception in noisy environments, this study aimed to evaluate the effects of a temporal processing-based auditory training program on improving speech perception in noisy conditions in adolescents with high functional ASD. Moreover, the effectiveness of the applied training program was evaluated after one month. 

## Materials & Methods

Participants

The study was conducted on 28 adolescents with high functional ASD (24 males and five females) with an age range of 10 to 16 years (±1.86). The participants were selected randomly from special schools and evaluated by clinicians. 

The inclusion criteria were having a normal hearing threshold (equal to or better than 25 dB in both ears at 250- 8000 Hz (ANSI2004)) and having no history of neurological disorders. All the subjects provided informed consent. In case of unwillingness of the participants or their parents, they were excluded from the study.

All the subjects had normal intelligence (IQ> 85) based on the Wechsler scale (15). The results of the test of acoustic and tympanometric reflexology of the middle ear were within the normal range (ear canal volume= 0.9-0.2 CM^3^; static compliance=0.3-1.5 mmHO; and sound pressure level=50 dapa) (16). Subjects with ASD were diagnosed according to the Diagnostic and Statistical Manual V criteria and based on a psychologist's or psychiatrist's diagnosis. Common factors for diagnosis of ASD include: 1) linguistic and social communication deficits; 2) repetitive and stereotypic patterns of movement and behavior; and 3) deficits in routine functions. These symptoms are generally observed in the early stages of growth (17-21).

 All the subjects were right-handed (based on the Persian version of the Edinburgh Questionnaire) and Persian language monolingual (22). 

The participants were randomly divided into two groups (an ASD group: 11 males and three females and a control group: 13 males and one female). Clinical history, baseline hearing, and verbal speech in noise were assessed using the Persian version of the monosyllabic words. 

The current study was approved by the Ethics Committee of the Social Welfare and Training Sciences University, Tehran, Iran (IR.USWR.REC.1395.317).

**Procedure**

The present study was performed in signal-to-noise ratios of 0 and +10, and scores were obtained in percentages (23, 24). The Speech ABR test was performed and analyzed using the Bio Logic Navigator Pro System. Right mastoid, forehead, and Cz silver chloride electrodes were used as reference, earth, and active electrodes, respectively. The speech stimulus /da/ with a 40 millisecond duration, that was previously used by Nina Kraus et al. in the Auditory Neuroscience Lab of the Northwestern University, was used in this study (14).

For the ASD group, in addition to the conventional training program (the routine program), training was performed based on temporal processing. However, the control group was only trained by the conventional training program. During training, stimuli were adapted based on each individual's prior response. In this study, the intervention focused on temporal-based bottom-up hearing processing using interval detection in noise and temporal pattern detection exercises. To perform these trainings, after obtaining the threshold for detecting the interval in noise, we began with the threshold at which each subject was able to detect the interval. The interval of silence in noise gradually decreased with each individual's progress. The score of each session was recorded and considered as the basis of training in next sessions. To do the duration pattern training, we increased intervals between the three pure 1000 Hz tones, and by attracting the attention of each subject, we asked them to repeat sounds that were different in the duration of brainstem evoked potential. As the patients progressed, intervals between the sounds decreased. The stimuli were provided at the easy level of hearing. The results of each session were recorded and compared with each individual's scores in the next sessions. The training program consisting of 30-minute sessions was conducted three times a week for six weeks (25). At the end of the six weeks of training, a speech perception test and the Speech ABR test were performed and the results were compared between the two groups. To evaluate the reliability of the outcomes, the assessments were repeated one month after the completion of the training (26). This study was conducted according to the rules of the Ethics Committee of the University of Social Welfare and Training. 

**Statistical analysis**

The normality of the data was first assessed using the Shapiro-Wilk test. A paired t-test was used for data analysis before and after the intervention to assess the data normality, and the covariance analysis test was used for the post-interventional results in the two groups.

A P-value less than 0.05 was considered statistically significant. All the statistical analyses were performed using the Statistical Package for the Social Sciences (SPSS) for Windows, version 10.

## Results

In this part, the descriptive features (mean and standard deviation) of the dependent variables were presented in two measurement steps including a pretest and a posttest for the control and experimental groups. 

Mean difference scores of speech perception in the presence of noise in the both signal-to-noise ratios of 0 and +10 were 60.0 and 74.0 in the intervention group after training and 41.71 and 53.71 in the control group, respectively. The scores also increased in the intervention group compared to the control group in the post-test. In the ASD group, speech perception in noise was significantly higher after the training than before the training. Moreover, there was a significant difference in the speech perception score before and after the training in the both signal-to-noise ratios of 0 and +10; however, there were no significant differences tin the control group (Table 1).

The latency of all the speech ABR waves was lower in the intervention group than in the control group. The mean latency of all waves in the two groups was similar before the implementation of the temporal-based training program, and there was no significant difference between the two groups (Table 2). Further, no significant difference was observed between the two groups in terms of wave amplitude. In addition, the results showed that the performance of the intervention group did not differ one month after the intervention (Table 3).

## Discussion

Individuals with ASD, despite their normal hearing ability, experience abnormal speech perception that is more pronounced in challenging environments and in the presence of competitive noise (27). In this study, speech perception increased in the intervention group and improvement in the scores was even observed one month after the intervention. Unfortunately, limited studies focused on improving speech perception of individuals with ASD, and this issue has so far been investigated only in one study . In a study by Rance et al., FM hearing aid was used to improve hearing and speech perception in people with ASD (28). Their results showed that this tool was effective in improving speech perception in noise (16.9% increase in the speech perception score; P-Value<0.001) and social interaction by increasing the signal-to-noise ratio. Our results showed a decrease in latencies of all the waves after the training compared to the pre-training phase and the control group. The reliability of the results was also observed one month after the training. This finding demonstrated that the training program had an impact on improving auditory temporal processing. 

Based on the previous findings and the findings of this study, in the filter class part of sABR, the delays of the initial waves and the final O wave were significantly reduced after completing the training program. This could indicate an improvement in synchronization of a neural response to recognition of the start and end of an auditory stimulus in individuals with ASD. In the source class part of sABR, reducing the latency of FFR components including D, E, and, E waves may indicate improvement in recognition of sudden components of FFR in the ASD group. This may improve the ability to detect consonants in this group. Thus, the training program used in this study is helpful in improving speech perception of ASD patients. 

Although few studies have examined the effects of bottom-up interventions by recording speech ABR in children with ASD, significant progress has been reported in various hearing processing disorders. Russo et al. (2010) evaluated the effect of the FFW training program on evoked auditory responses of the brainstem in five children with ASD. They found a reduced latency of the initial waves and more suitable speech processing at the brainstem level (29). In a study by Krishnamun (2013) using the FFW program in two children with auditory processing disorder (APD), the reduction of speech ABR latency after the intervention was considered to be associated with neural plasticity at the brainstem level (30). Fillippini (2012) in their study used Speech ABR in silent and noisy environments and performed a training program for eight weeks in APD children. They reported the effect of the training program on reducing the latency of the initial waves (31). In a similar study, Russo et al. (2005) used the Aerobic Computer Rehab program in children with a relatively large range of linguistic problems. According to their findings, Speech ABR waves were more pronounced in the encoding of linguistic features of the /da/ stimulant in the presence of background noise (32). Although these studies have been conducted mainly on APD, their results are in line with ours. 

Our findings are in line with previously published studies, although they were conducted mainly on other APDs or used other methods of rehabilitation. However, according to our knowledge, no study was carried out to improve speech perception in patients with ASD, and this is the first study attempting to improve speech perception by considering auditory processing, due to the major role of auditory processing in speech perception. 

We investigated the effects of a bottom-up training program on brainstem neural plasticity and speech perception improvement in noisy environments. In this case, although the results were clear, we suggest to conduct more detailed studies to further elucidate the impact of this training program.

**Table 1:** Results of the speech perception test in noise in the two signal-to-noise ratios of 0 and +10 for the ASD and control groups before and after the training

P-value*	(14=n )Intervention group		(14=n )Control group		Variables
	**Postest**	**Pretest**	**Posttest**	**Pretest**	
<0.001	7,01±60,0	9,09±45,49	41.71±5.36	40,57±8,58	**Signal to noise ratio of zero**
<0.001	74.0±6.64	56,28±6,92	53.71±6.78	52,58±5,84	**Signal to noise ratio of +10**

*^ANCOVA^

**Table 2:** The mean and standard deviation of speech ABR latency in the both groups after the training

Speech ABR latency (ms**)**	**Control group (n=14)**		**Intervention group (n=14)**		P-value*
	**Posttest**	**pretest**	**posttest**	**Pretest**	
V	6,88±0,40	6.46±0.39	6,98±0,58	6,90±0,60	0,01
A	7,78±0,32	7.38±0.53	7,86±0,51	7,56±0.78	0.02
C	20,08±0,64	19.06±0.77	20,80±1,51	19,94±0.92	0,001
D	24,43±0,88	23.14±1.11	27,56±1,42	24,78±1,15	<0,001
E	33,41±1,21	31.52±0.50	33,72±1,70	33,29±1,59	<0,001
F	40,89±0,94	40.09±0.71	41,84±1,66	41,35±0,96	0.002
O	49,53±0,90	48.27±0.56	50.36±1,86	49,58±0,96	<0,001

*Ancova

**Table 3.** The mean and standard deviation of speech ABR latency in the intervention group immediately after the training and one mouth later

Speech ABR latency	Intervention group (n=14)		Intervention group (n=14)	P-value*
	**Post-training**		**Follow up**	
V	6.46±0.39		6.47±0.39	0.47^**^
A	7.38±0.53		7.41±0.51	0.16^*^
C	19.06±0.77		19.06±0.82	0.17^*^
D	23.14±1.11		23.13±0.50	0.78^**^
E	31.52±0.50		31.35±0.60	0.11^**^
F	40.09±0.71		40.05±0.63	0.68^**^
O	48.27±0.56		48.40±0.46	0.17^*^

*t test

** Wilcoxon

## In Conclusions,

Improvement in speech perception in noisy environments and latency of speech ABR waves immediately after performing the training program and one month later suggests the role of temporal auditory processing in brainstem bottom-up pathways in speech perception.

Speech perception improvement in noise and a reduction in ABR latency immediately after performing temporal training and one month later suggest that auditory temporal processing has a critical role in the brainstem (bottom-up) processing of speech.

## References

[1] Samsam M, Ahangari R, Naser SA (2014). Pathophysiology of autism spectrum disorders: Revisiting gastrointestinal involvement and immune imbalance. World J Gastroenterolog : WJG.

[2] Anderson S, Kraus N (2010). Objective neural indices of speech-in-noise perception. Trends amplification.

[3] Toplak ME, Dockstader C, Tannock R (2006). Temporal information processing in ADHD: findings to date and new methods. Journal of neuroscience methods.

[4] Poldrack RA, Temple E, Protopapas A, Nagarajan S, Tallal P, Merzenich MM (2001). Relations between the neural bases of dynamic auditory processing and phonological processing: evidence from fMRI. J Cogn Neurosci.

[5] Rosen S (1992). Temporal information in speech: acoustic, auditory and linguistic aspects. Philosophical Transactions of the Royal Society of London B: Biological Sciences.

[6] Alcantara JI, Cope TE, Cope W, Weisblatt EJ (2012). Auditory temporal-envelope processing in high-functioning children with Autism Spectrum Disorder. Neuropsychologia.

[7] de Boer J, Thornton AR (2008). Neural correlates of perceptual learning in the auditory brainstem: efferent activity predicts and reflects improvement at a speech-in-noise discrimination task. J neurosci.

[8] Song JH, Skoe E, Wong PC, Kraus N (2008). Plasticity in the adult human auditory brainstem following short-term linguistic training. cogn neurosci.

[9] Tallal P, Stark RE (1981). Speech acoustic-cue discrimination abilities of normally developing and language-impaired children. J Acoust Soc Am.

[10] Merzenich MM, Jenkins WM, Johnston P, Schreiner C, Miller SL, Tallal P (1996). Temporal Processing Deficits of Language-Learning Impaired Children Ameliorated by Training. Science.

[11] Anderson S, Skoe E, Chandrasekaran B, Kraus N (2010). Neural timing is linked to speech perception in noise. JNeurosci.

[12] Galbraith GC, Arbagey PW, Branski R, Comerci N, Rector PM (1995). Intelligible speech encoded in the human brain stem frequency-following response. Neuroreport.

[13] Johnson KL, Nicol TG, Kraus N (2005). Brain stem response to speech: a biological marker of auditory processing. Ear hear.

[14] Kraus N, Nicol T (2005). Brainstem origins for cortical 'what' and 'where' pathways in the auditory system. Trends neurosci.

[15] Bruce G (2004). The Wechsler Preschool and Primary Scale of Intelligence, Third Edition (WPPSI-III) San Antonio, TX: The Psychological Corporation. Canadian Journal of School Psychology.

[16] Katz J, Burkard RF, Medwetsky L (2002). Handbook of Clinical Audiology.

[17] Rapin I, Dunn M (2003). Update on the language disorders of individuals on the autistic spectrum. Brain & development.

[18] Siegal M, Blades M (2003). Language and auditory processing in autism. Trends in cognitive sciences.

[19] Tager-Flusberg H, Caronna E (2007). Language disorders: autism and other pervasive developmental disorders. Pediatric Clinics.

[20] Rapin I, Dunn M (2003). Update on the language disorders of individuals on the autistic spectrum. Brain & development.

[21] Siegal M, Blades M (2003). Language and auditory processing in autism. Trends in cognitive sciences.

[22] Oldfield RC (1971). The assessment and analysis of handedness: the Edinburgh inventory. Neuropsychologia.

[23] Mosleh M (2001). Development and Evaluation of a Speech Recognition Test for Persian Speaking Adults. Audiology.

[24] Omidvar S, Jafari Z, Tahaei AA, Salehi M (2013). Comparison of auditory temporal resolution between monolingual Persian and bilingual Turkish-Persian individuals. International Journal of Audiology.

[25] Weihing J, Chermak GD, Musiek FE (2015). Auditory Training for Central Auditory Processing Disorder. Seminars in Hearing.

[26] Chermak GD, Musiek FE (2013). Handbook of Central Auditory Processing Disorder, Volume II, Second Edition: Comprehensive Intervention: Plural Publishing, Incorporated.

[27] Lepistö T, Kuitunen A, Sussman E, Saalasti S, Jansson-Verkasalo E, Nieminen-von Wendt T (2009). Auditory stream segregation in children with Asperger syndrome. Biological psychology.

[28] Rance G, Saunders K, Carew P, Johansson M, Tan J (2014). The use of listening devices to ameliorate auditory deficit in children with autism. J peds.

[29] Russo NM, Hornickel J, Nicol T, Zecker S, Kraus N (2010). Biological changes in auditory function following training in children with autism spectrum disorders. Behav Brain Funct.

[30] Krishnamurti S, Forrester J, Rutledge C, Holmes GW (2013). A case study of the changes in the speech-evoked auditory brainstem response associated with auditory training in children with auditory processing disorders. International journal of pediatric otorhinolaryngology.

[31] Filippini R, Befi-Lopes DM, Schochat E (2012). Efficacy of auditory training using the auditory brainstem response to complex sounds: auditory processing disorder and specific language impairment. Folia Phoniatr Logop.

[32] Russo NM, Nicol TG, Zecker SG, Hayes EA, Kraus N (2005). Auditory training improves neural timing in the human brainstem. Behav Brain Res.

